# High-Accuracy Polymer Property Detection via Pareto-Optimized SMILES-Based Deep Learning

**DOI:** 10.3390/polym17131801

**Published:** 2025-06-28

**Authors:** Mohammad Anwar Parvez, Ibrahim M. Mehedi

**Affiliations:** 1 Department of Chemical Engineering, College of Engineering, King Faisal University, Al-Ahsa 31982, Saudi Arabia; 2 School of Robotics, XJTLU Entrepreneur College (Taicang), Xi’an Jiaotong-Liverpool University, No. 111 Taicang Ave., Taicang, Suzhou 215400, China

**Keywords:** polymer property detection, pareto optimization algorithm, Molecular Input Line Entry System, linear scaling normalization, hybrid deep learning

## Abstract

Polymers have a wide range of applications in materials science, chemistry, and biomedical domains. Conventional design methods for polymers are mostly event-oriented, directed by intuition, experience, and abstract insights. Nevertheless, they have been effectively utilized to determine several essential materials; these techniques are facing important challenges owing to the great requirement of original materials and the huge design area of organic polymers and molecules. Enhanced and inverse materials design is the best solution to these challenges. With developments in high-performing calculations, artificial intelligence (AI) (particularly Deep learning (DL) and Machine learning (ML))-aided materials design is developing as a promising tool to show development in various domains of materials science and engineering. Several ML and DL methods are established to perform well for polymer classification and detection presently. In this paper, we design and develop a Simplified Molecular Input Line Entry System Based Polymer Property Detection and Classification Using Pareto Optimization Algorithm (SMILES-PPDCPOA) model. This study presents a novel deep learning framework tailored for polymer property classification using SMILES input. By integrating a one-dimensional convolutional neural network (1DCNN) with a gated recurrent unit (GRU) and optimizing the model via Pareto Optimization, the SMILES-PPDCPOA model demonstrates superior classification accuracy and generalization. Unlike existing methods, our model is designed to capture both local substructures and long-range chemical dependencies, offering a scalable and domain-specific solution for polymer informatics. Furthermore, the proposed SMILES-PPDCPOA model executes a one-dimensional convolutional neural network and gated recurrent unit (1DCNN-GRU) technique for the classification process. Finally, the Pareto optimization algorithm (POA) adjusts the hyperparameter values of the 1DCNN-GRU algorithm optimally and results in greater classification performance. Results on a benchmark dataset show that SMILES-PPDCPOA achieves an average classification accuracy of 98.66% (70% Training, 30% Testing) across eight polymer property classes, with high precision and recall metrics. Additionally, it demonstrates superior computational efficiency, completing tasks in 4.97 s, outperforming other established methods such as GCN-LR and ECFP-NN. The experimental validation highlights the potential of SMILES-PPDCPOA in polymer property classification, making it a promising approach for materials science and engineering. The simulation result highlighted the improvement of the SMILES-PPDCPOA system when compared to other existing techniques.

## 1. Introduction

Polymers are the most significant kind of materials in industries and daily use. In the past several years, polymers have been searched for a huge variety of applications from everyday existence to frontier technology like building, energy, food industry, aerospace, and medicine [1]. Owing to the wide range of possible and existing industrial usages, the requirement for novel polymer materials with purpose-designed assets is important. Nevertheless, due to the inexhaustibility of chemical space, polymers hold a group of distinctive electrical, chemical, and physical features [2]. The integration of wide-ranging chemical compounds, intricate polymer chain arrangements, diverse monomer frameworks, and numerous synthesis models brings immense opportunities together with trouble in polymer selection and production [3]. For investigators, with high-dimensional polymer data and a huge number of published articles, it is source-intensive to screen the declared information and remove valuable data on structure-property relations [4].

Polymeric materials are among the rapidly progressive entities. A huge amount of polymers with remarkable features are integrated, and efforts are being performed on the production of more predetermined and desired features [5]. The industrial application of polymeric materials comprises but is not restricted to the aerospace, automotive industry, building, packaging, composites, construction, textiles, and electronics [6]. Identification and characterization of polymers is another field, which is the concept of recent investigation that needs several instruments and analytical models. Recognition of polymeric materials primarily depends on their physico-chemical, mechanical, thermal, and viscoelastic properties that result in the origination of models like microscopy, spectroscopy, thermal analysis, and more [7]. Conventionally, Artificial Intelligence (AI) methods for polymer design have been particularly considered [8]. Deep learning (DL) and Machine learning (ML) have been applied to various aspects of chemistry and material sciences by supplying effective models of utilizing data that allow timely prognosis of assets, the route of synthesis, and the invention of unique molecules [9]. The utilization of DL and ML in polymer assets, especially for classification and detection, has proved its potential rapidly in the modern world [10]. While many essential physical properties, such as elastic modulus, density, and glass transition temperature, are crucial to polymer performance, our present model focuses on classifying polymers based on those properties for which annotated SMILES-based datasets are currently available, including bandgap, dielectric constant, and refractive index. Future work will expand the model’s capability to predict additional mechanical and thermal properties as relevant data become accessible.

In this paper, we design and develop a Simplified Molecular Input Line Entry System Based Polymer Property Detection and Classification Using Pareto Optimization Algorithm (SMILES-PPDCPOA) model. In the initial stage, the data pre-processing employs linear scaling normalization (LSN) for transforming input data into a suitable format for analysis. Furthermore, the proposed SMILES-PPDCPOA model executes a one-dimensional convolutional neural network and gated recurrent unit (1DCNN-GRU) technique for the classification process. Finally, the Pareto optimization algorithm (POA) adjusts the hyperparameter values of the 1DCNN-GRU algorithm optimally and results in greater classification performance. The simulation validation of the SMILES-PPDCPOA algorithm can be tested on a benchmark dataset, and the outcomes are measured for various measures. The contributions of the SMILES-PPDCPOA model in the field of polymer property detection and classification can be summarized as follows:

The SMILES-PPDCPOA model significantly improves the process of detecting and classifying polymer properties, addressing the limitations of conventional design methods that rely heavily on intuition and experience.

By integrating a one-dimensional convolutional neural network and gated recurrent unit (1DCNN-GRU) methodology, the model leverages advanced deep learning techniques to enhance classification accuracy.

The incorporation of the Pareto optimization algorithm (POA) for hyperparameter tuning allows for optimal adjustments, leading to improved performance in classification tasks.

The model achieves an impressive average classification accuracy of 98.66% across eight polymer property classes, showcasing its effectiveness compared to traditional methods.

By showcasing the potential of AI, particularly deep learning and machine learning, in the field of polymer science, the SMILES-PPDCPOA model contributes to the broader movement towards enhanced and inverse materials design.

These contributions highlight the significance of the SMILES-PPDCPOA model in advancing the field of polymer science and engineering through innovative methodologies and high-performance outcomes. Section 2 depicts the literature survey of existing methods and their limitations, Section 3 details the materials and methods, and the dataset used to train and test the SMILES-PPDCPOA model is described. It describes data preparation, including linear scaling normalization (LSN), and the classification architecture of the one-dimensional convolutional neural network and gated recurrent unit (1DCNN-GRU). It also discusses how hyperparameter adjustment with the Pareto optimization method (POA) improves model performance. Section 4 analyses the proposed work with validation as SMILES-PPDCPOA model performance. It compares classification accuracy, precision, recall, and computing efficiency to other approaches. Detailed benchmark dataset results demonstrate the model’s polymer property classification accuracy, and Section 5 summarizes the study results and highlights the SMILES-PPDCPOA model’s polymer property categorization benefits. It considers materials science and engineering consequences and suggests polymer design and categorization research paths.

## 2. Literature of Works

The field of polymer science has witnessed significant advancements in recent years, particularly in the development of novel materials and their applications across various domains. This literature survey aims to provide a comprehensive overview of recent studies that highlight key developments in polymer technology, focusing on pH-sensitive polymers, conductive hydrogels, machine learning applications in polymer property prediction, and advancements in nanomedicine. In [11], an ensemble DL method is proposed for the automated recognition of nanoparticles. The major part of previous research on nanoparticle recognition and classification of fixed instances depends on transmission electron microscopy (TEM) images or scanning electron microscopy (SEM), which is critical to biological imaging. This paper fine-tunes a DNN structure, YOLOv8, on a precisely annotated database of co-related optical and SEM images. Abhishek and Raghukiran [12] developed a contactless model employing deep classification to measure and detect shape variations in responsive polymers. The deep network application in responsive structural element recognition has not been attempted before. The shape variations are related to diverse deformation heights, lengths, or angles over an innovative formula-based correlation. Song et al. [13] projected a model for classifying and identifying surface flaws in polyimide foam depending on upgraded GoogLeNet, intending to accurately and quickly identify and detect surface flaws in foam products. By leveraging the Inception blocks, presenting the ECA attention mechanism, and increasing an LSTM system, generalizability and precision are effectively improved.

Mohsenzadeh et al. [14] introduced an automated and accurate model for particle detection and detailed mapping of particle positions in SEM micrographs by incorporating a deep CNN with sophisticated image processing models. After succeeding in particle recognition, dual dispersion features are presented, such as supercritical clustering and size uniformity, to evaluate the effect of particle dispersion on features. An adapted micromechanical technique is utilized to evaluate the nanocomposite thickness and interfacial strength. In [15], the automated solar defect classification and detection are presented by applying DL. The projected method contains three stages: Reduction and Feature Extraction, Classification, and Pre-processing. CNN-based LSTM and reduction and feature extraction for the solar defect classification are employed. In the pre-processing stage, the distortion correction model is presented to extract the distortions by applying a special class of Gaussian filtering and enhancing the dissimilarity. This work improves the present CNN-based feature extractor method by integrating Wavelet Transform (WT) for enhanced feature representation and implementing Principal Component Analysis (PCA) for reducing features.

Azad et al. [16] projected a DL-based method for the independent microscopic damage evaluation of FRPs. Multiple computationally effective pre-trained DL techniques like NasNet Mobile, DenseNet121, MobileNet, and EfficientNet were assessed for their implementation to recognize diverse damage modes independently, consequently decreasing the requirement for manual interpretation. Lim et al. [17] developed PINN that associates experimental data with the deduced physical knowledge described by Fourier’s law of heat diffusion to process thermographic data. With the assistance of PINN, nonuniform conditions are evaluated and extracted from the original thermograms, emphasizing the defect information. Then, principal component thermography (PCT) is employed to minimize size and remove features from the processed thermograms. Additionally, PINN can evaluate unknown physical parameters like the material’s thermal diffusivity. Ofridam et al. [1] classified pH-sensitive polymers and explored their potential applications in various fields, including drug delivery and biosensing. These polymers exhibit a change in their physical or chemical properties in response to pH variations, making them suitable for targeted therapies. The study emphasizes the versatility of pH-sensitive materials and their ability to enhance the efficacy of therapeutic agents by ensuring localized delivery.

Zhu et al. [2] provided a thorough review of conductive hydrogels, discussing their classifications, properties, and applications. Conductive hydrogels are emerging as vital materials in soft electronics, bioelectronics, and wearable devices due to their flexibility, biocompatibility, and electrical conductivity. The authors highlighted the importance of material composition and structural design in optimizing the performance of these hydrogels for specific applications. The integration of machine learning techniques in polymer science has opened new avenues for property prediction and material design. Park et al. [18] demonstrated the use of graph convolutional networks to predict and interpret polymer properties effectively. Their findings suggest that machine learning models can significantly reduce the time and resources required for experimental validation, leading to more efficient material development processes. Sharma et al. [7] further expanded on this theme by discussing advances in computational intelligence for polymer composite materials. Their study focused on machine learning-assisted modeling, analysis, and design, emphasizing the potential of these technologies to enhance the understanding of complex polymer behaviors and facilitate the development of high-performance composite materials. Kim et al. [9] reviewed post-synthetic modifications in porous organic polymers, particularly for biomedical applications. The study highlighted how these modifications can tailor the properties of polymers for specific biomedical uses, such as drug delivery and tissue engineering. The ability to customize polymer characteristics is crucial for improving biocompatibility and functionality in medical applications.

Li et al. [4] explored the development of dendritic polymer-based nanomedicines for cancer diagnosis. Their work emphasizes the role of dendritic polymers in enhancing the efficacy of diagnostic tools, showcasing their potential to improve early detection and treatment outcomes in cancer care. Azad et al. [16] investigated the application of deep learning techniques for microscopic damage assessment in fiber-reinforced polymer composites. Their research illustrates the potential of advanced computational methods to analyze and predict damage in composite materials, which is crucial for ensuring the reliability and safety of polymer-based structures in various engineering applications. Despite the advancement of AI-driven techniques in materials science, polymer property detection and classification remain challenging due to the complexity and vastness of the chemical space involved. Current methods often suffer from limited scalability, reliance on manual feature engineering, and suboptimal classification performance. Moreover, few models integrate optimization techniques like Pareto Optimization to fine-tune the hyperparameters of deep learning models for such tasks. This research addresses these gaps by proposing a fully integrated solution that combines data normalization, deep learning techniques, and optimization algorithms, offering a significant leap forward in polymer property detection.

## 3. Materials and Methods

**Figure 1:**
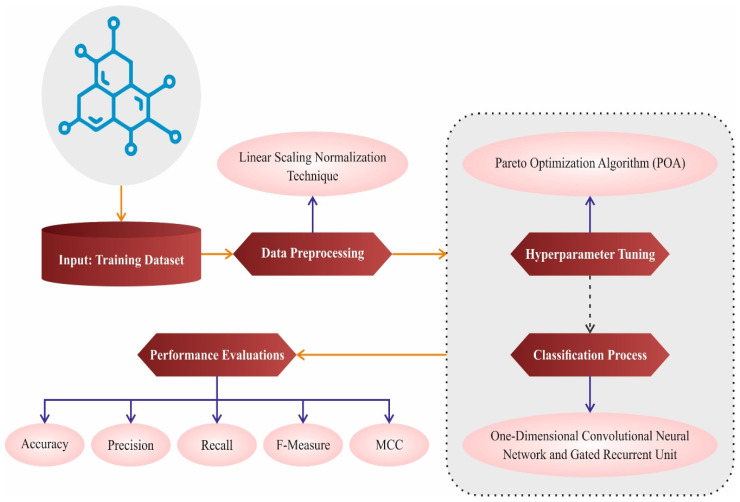
Overall workflow of the SMILES-PPDCPOA algorithm.

In this paper, we design and develop a SMILES-PPDCPOA model. The proposed SMILES-PPDCPOA technique relies on enhancing the detection and classification process of polymer properties. To achieve that, the proposed SMILES-PPDCPOA algorithm involves data pre-processing, classification, and hyperparameter tuning stages. Figure 1 represents the overall workflow of the SMILES-PPDCPOA technique. The neural network for the SMILES-PPDCPOA model was developed using the TensorFlow and Keras libraries in Python (TensorFlow 2.19.0, Keras 3.10.0). These frameworks provided flexibility in model architecture design and allowed integration with custom optimization routines for Pareto Optimization.

### 3.1. LSN-Based Data Pre-Processing

In the initial stage, the data pre-processing employs LSN for transforming input data into a suitable format for analysis. Linear Scaling Normalization (LSN) is a data pre-processing method utilized to normalize the feature range, particularly when the data diverges extensively [19]. As part of polymer property classification and detection, LSN has been employed to convert arithmetic properties like thermal conductivity, tensile strength, or elasticity to a reliable scale, normally between (0, 1). This assists the ML method in understanding the data more efficiently by stopping features with a wider choice from affecting the performance of the model. By standardizing the data, LSN guarantees that all features correspond to the classification task. This may improve the stability and accuracy of predictive methods, making them more consistent in identifying and classifying polymer properties. The dataset employed in this study consists of 6265 records spanning eight distinct polymer property classes: Atomization Energy (EAT), Crystallization Tendency (XC), Bandgap (chain and bulk—EGC, EGB), Electron Affinity (EEA), Ionization Energy (EI), Refractive Index (NC), and Dielectric Constant (EPS). These properties were selected because they represent critical chemical and physical features that influence polymer functionality in real-world applications. We acknowledge that potential class imbalance or selection bias may affect model performance. To address this, we verified that none of the classes disproportionately dominated the dataset, and we applied balanced accuracy, precision, recall, and F1-score metrics during evaluation to ensure fairness across classes. To handle incomplete and noisy data, the dataset underwent rigorous pre-processing:

- Missing values were imputed using the class-wise mean for numeric fields.
- Input data was normalized using Linear Scaling Normalization (LSN).
- Dropout regularization (rate = 0.2) and early stopping were applied during training to prevent overfitting.

Furthermore, the GRU component of the hybrid model showed resilience to noise due to its gated memory structure, which allowed it to retain relevant features over time while filtering out noisy inputs.

The SMILES string encodes the polymer’s chemical structure in a linear sequence format. The 1DCNN component captures local chemical features (e.g., functional groups), while the GRU layer processes longer-range dependencies across the polymer backbone. This hybrid architecture enables the model to learn complex structure–property relationships without manual feature engineering or reliance on 3D molecular descriptors.

### 3.2. DCNN-GRU-Based Classification Model

Furthermore, the proposed SMILES-PPDCPOA model executes the 1DCNN-GRU technique for the classification process. Recently, CNNs, which are the most traditional DL networks, have captivated a lot of attention owing to their outstanding feature extraction and great fitting capability [20]. However, CNNs have established significant ability in computer vision (CV) tasks such as image classification, but we have problems in directly classifying 1D data. Numerous studies tried to transform 1D data into 2D images by applying various methods, allowing CNNs to classify 1D data. Nevertheless, this model frequently results in robust computing. To successfully deal with the above problems, investigators presented that classification and feature extraction are implemented straightforwardly on 1D data utilizing ID-CNN. The conventional ID-CNN has 5 major elements: a fully connected (FC) layer, a pooling layer, a convolutional layer, a Softmax function output layer, and an activation layer. The convolutional kernel is learned in the convolutional layer to carry out convolutional processes on the input data to gain feature mapping. Owing to the features of local connectivity and weight sharing in convolutional layers, the system can effectively take local dependencies inside the data. The principle of the convolution process is as shown:

(1) $$y^{L}=\sum_{i=1}^{c^{L-1}}w_{i,c}^{L}⊗x_{i}^{L-1}+b_{i}^{L}$$

Whereas the layer output L is $y^{L},\;x_{i}^{L-1}$ characterizes the layer L−1 output of the i th channel, $c^{L-1}$ refers to c th the layer L−1 channel, $w_{i,c}^{L}$ signifies the weighted matrix of the L-layer convolution kernel, $b_{i}^{L}$ means bias, and ⊗ symbolizes a convolutional calculation. Afterward, the activation function has been applied to nonlinearly transform the output features of the convolution layer. The activation function of the Rectified Linear Unit (ReLU) is a common option and can be described as demonstrated:

(2) $$a^{L(i,j)}=ReLU\left(x^{L\left(i,j\right)}\right)=max\left(x^{L\left(i,j\right)},\;0\right)\;\;$$

Whereas $x^{L(i,j)}$ signifies jth feature value in the ith feature maps of the layer L and $a^{L(i,j)}$ characterizes the consistent activation value, ReLU represents the activation function. The pooling layer decreases the feature mapping dimensionality, thus decreasing the parameter counts and computational efficiency of the following layers while preserving significant features. Considering that maximal pooling exceeds average pooling in successfully managing 1D series tasks, this study utilizes maximal pooling.

(3) $$y^{L(i,j)}=\underset{(j-1)S+1<t<jS}{max}\left\{X^{L\left(i,t\right)}\right\}$$

Whereas $x^{L(i,t)}$ denotes a value of the t th neuron in the i th channel of layer L. The pooling kernel dimensions were represented by S. The output value of the j th neuron of the i th channel in layer L is signified by $y^{L\left(i,j\right)}$. The layer of FC is the main portion of CNNs that flattens the removed deep features by the pooling and convolutional layers to 1D vectors and classifies the removed outcomes by the layer of Softmax. The equation for the FC layer is as demonstrated:

(4) $$y^{L+1(j)}=\sum_{i=1}^{n}w_{ij}^{L}x^{L(i)}+b_{j}^{L},p(x{)}_{i}=\frac{e^{z_{i}}}{\sum_{k=1}^{c}e^{z_{k}}},\;for\;i=1,2,\ldots ,C$$

**Figure 2:**
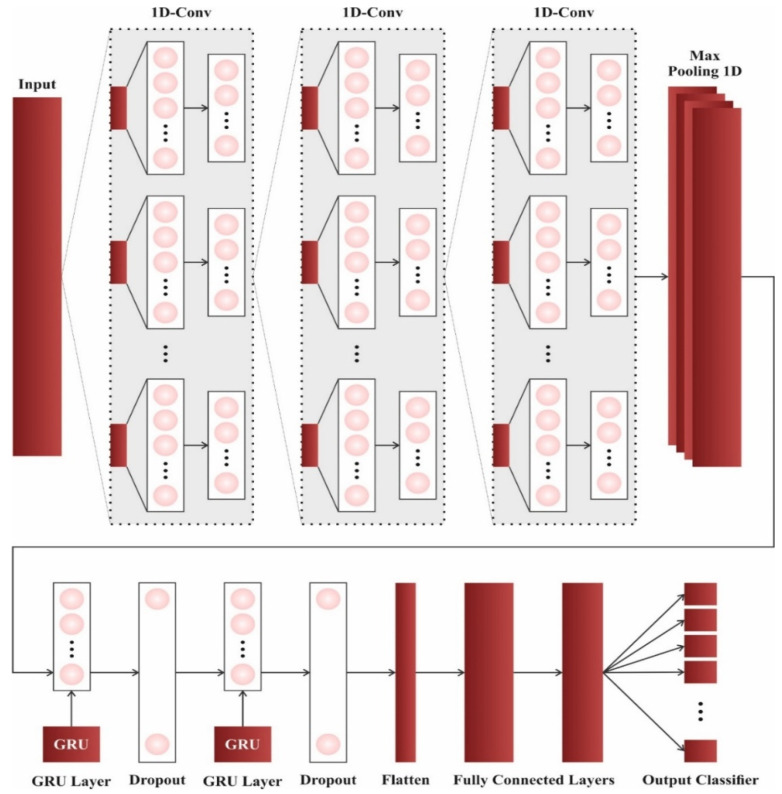
Structure of the 1DCNN-GRU technique.

Whereas $x^{L(i)}$ denotes the output value of the layer L; the weighting of the i th neuron in layer and the j th neuron in layer L+1 are signified as $w_{ij}^{L};\;b_{j}^{L}$ symbolizes the bias of each neuron of layer L to the j th neuron of layer L+1; C designates the sum of categories; $z_{i}$ symbolizes the value in the i th deactivated neuron within the output layer; and the output layer i th probability output of the neuron is represented by $p(x{)}_{i}.$ Feed-forward neural networks provide just a static mapping of outputs and inputs, reducing their applicability to static classification tasks. To deal with the needs of temporal prediction tasks, such as natural language processing (NLP) and speech recognition, numerous studies have focused on the growth of recurrent neural networks (RNNs). Nevertheless, challenges such as gradient explosion weaken the RNN’s performance in handling time series tasks. To deal with these challenges, LSTM was presented, substituting the RNN’s HL with a memory block able to retain previous data. Later, we presented a GRU-based LSTM. Therefore, the GRU establishes performance similar to that of LSTM, whereas demanding smaller training parameters and presenting a fast training method. Figure 2 portrays the structure of the 1DCNN-GRU technique. The hybrid 1DCNN-GRU architecture was chosen due to its ability to capture both spatial patterns (via 1D-CNN) and temporal dependencies (via GRU) inherent in the SMILES-based input features. 1DCNN extracts localized feature interactions across the molecular descriptor sequences, while GRUs are capable of learning longer-range dependencies without the complexity of LSTM.

- 1DCNN Layers:
  ▪ 2 convolutional layers
  ▪ Filter sizes: 64 and 128
  ▪ Kernel size: 3
  ▪ Activation: ReLU
  ▪ MaxPooling layer after each convolution
- GRU Layers:
  ▪ 1 GRU layer
  ▪ Hidden units: 128
  ▪ Dropout: 0.2
- Fully Connected Layers:
  ▪ 2 Dense layers with 64 and 8 units, respectively
  ▪ Output layer with Softmax activation

This combination was selected based on the prior literature showing its effectiveness in sequential classification tasks, and it was found empirically superior to other combinations (e.g., CNN-LSTM, GRU-only) in our experiments.

(5) $$R_{t}=\sigma \left(W_{r}X_{t}+U_{r}H_{t-1}\right)\;\;$$

(6) $$Z_{t}=\sigma \left(W_{z}X_{t}+U_{z}H_{t-1}\right)\;$$

(7) $${\bar{H}}_{t}=tanh\left(\left(W_{H}X_{t}\right)+U_{H}\left(R_{t}⊙H_{t-1}\right)\right)$$

(8) $$H_{t}=\left(Z_{t}⊙H_{t-1}\right)+\left(\left(1-Z_{t}\right)⊙{\bar{H}}_{t}\right)$$

Here, $Z_{t}$ and $R_{t}$ embodies update and reset gates, $W_{r}$ and $W_{z}$ denotes weights linked to the input vector, $U_{r}$ and $U_{z}$ are connected to the weights of the prior hidden state, $H_{t-1}$ and $H_{t}$ refers to the output at time t−1 and t, and ⊙ means product.

### 3.3. POA-Based Hyperparameter Tuning Process

Finally, the POA adjusts the hyperparameter values of the 1DCNN-GRU algorithm optimally and results in greater classification performance. Pareto optimization is a multi-objective optimization model that searches for the optimal exchange among contrasting targets [21]. As part of neural network (NN) parameter updating and training, POA has been applied to discover the optimal collection of hyperparameters, which concurrently enhance various performance metrics of the NN. The initial phase in POA describes the objective functions, signifying the dissimilar performance metrics to improve the parameters. The objective function can be described as $f(x)=\left\{f_{1}\left(x\right),\;f_{2}\left(x\right),\;\ldots ,\;f_{k}\left(x\right)\right\}$. The hyperparameter search space was defined based on the prior literature and empirical tuning. The ranges were:

- Learning rate: [0.0001, 0.01]
- Number of GRU units: [64, 128, 256]
- Number of filters in CNN: [32, 64, 128]
- Dropout rate: [0.1, 0.5]
- Batch size: [32, 64, 128]

These ranges were selected to balance computational efficiency and model accuracy. POA iteratively refined these values to converge on the optimal configuration.

Now, x refers to the decision variable, and the k-value characterizes the target amount. f(x) is expressed to attain Equation (9).

(9) $$J_{1}=\frac{1}{N}\sum (y-\hat{y}{)}^{2}$$

(10) $$J_{2}=\frac{\left(N_{weight}+N_{bias}\right)}{N_{Params}}$$

During Equation (9) $J_{1}$ is described as the mean-square error of (y) from the computed value y, N can be signified as the sample counts, and the weighting counts in the method are represented as $N_{weight}.$ $N_{bias}$ denotes the channel’s distortion quantity, however $N_{Params}$ stands for a total quantity of design variables. Based on the parameter, the model complexity (J) is calculated utilizing Equation (10). Computing difficulty by separating the total number of variables by the total sample count. Hence, the POA is applied to identify the optimal solution while identifying the searching region. Formerly, the search area value counts the hyperparameters that might yield. The search area might be hybrid, discontinuous, or continuous. Certain variables, which may be combined into the searching region, contain the learning model, the hidden layer counts, the neuron counts inside all layers, and the parameters of regularization. The second stage is to offer possible solutions for a dissimilar collection of hyperparameters. Panel search or random selection is a model frequently utilized for building a pool of possible replies from the searching region. The candidate dominance solution has been applied in that dual solutions are compared with the dominance features. When the solution $x_{1}$ directs other solutions $x_{2}$, and once the solution is superior then $x_{2}$ in a relatively single objective function and is poorer in some other function of the objective, then the dominance solution can be described as in Equations (11) and (12): $x_{1}$ is dominated $x_{2}$ and only if,

(11) $$f_{i}(x_{1})\leq f_{i}(x_{2})\;for\;all\;j=1,2,\ldots ,k\;\;$$

(12) $$f_{j}\left(x_{1}\right)<f_{j}\left(x_{2}\right)\;for\;one\;at\;least\;j=1,2,\ldots ,k\;$$

Pareto optimality is if no other option solution in the searching region outshines the specified solutions, signified by x. It might be expressed mathematically: a response x is optimal on condition that there is no other solution $x^{\prime }$ in the searching region like $x^{\prime }$ dominated x. The front of Pareto gathers each of the optimal promising outcomes from the search. It characterizes the best exchange among the inconsistent objective functions described in Equation (13).

(13) $$PF=\left\{x|x\;is\;Pareto\;optimal\right\}\;\;$$

The NN has been trained to utilize the objective function for all candidate solutions. The hyperparameters of the NN are upgraded in training utilizing an optimizer model like stochastic gradient descent. The efficiency of the NN on all performance indicators is established after training is finished for all possible solutions by assessing the objective functions. The following phase utilizes a POA to discover the optimum incorporation of hyperparameters for the maximization of each related indicator of performance. The POA captures an evolutionary model, altering and merging preceding solutions to progress novel candidates for optimization. The model aims to find a set of non-dominant solutions, which cannot be produced well in certain metrics without negatively affecting the other performances. The best hyperparameters are selected from amongst the non-dominant choices, and finally, the POA. The POA model originates a fitness function (FF) to reach boosted performance of classification. It outlines an optimistic number to embody the better outcome of the candidate solution. In this paper, the minimization of the classification rate of error was reflected as FF. Its mathematical formulation is represented in Equation (14).

(14) $$fitness\left(x_{i}\right)=ClassifierErrorRate\left(x_{i}\right)\;\;\;\;\;\;\;\;\;\;\;\;\;\;\;=\frac{number\;of\;misclassified\;samples}{Total\;number\;of\;samples}*100$$

## 4. Performance Validation

Here, the experimental analysis of the SMILES-PPDCPOA system is verified under the dataset [22]. The dataset contains 6265 records under eight class labels as exposed in Table 1.

**Figure 3:**
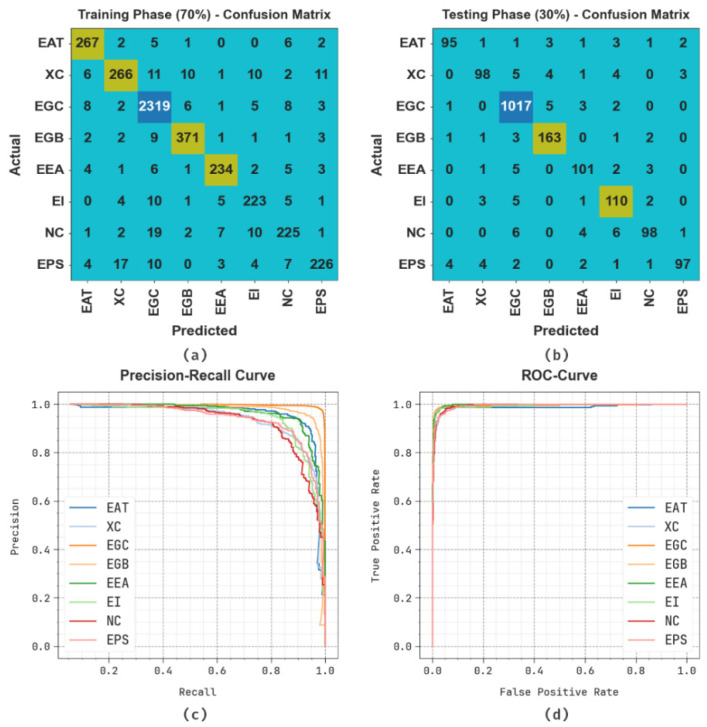
(**a**,**b**) 70%TRPH and 30%TSPH of the confusion matrix and (**c**,**d**) curves of PR and ROC.

Figure 3 represents the classifier result of the SMILES-PPDCPOA algorithm. Figure 3a,b displays the confusion matrix with correct recognition and classification of every class below 70%TRPH and 30%TSPH. Figure 3c establishes the PR analysis, representing superior performance across each class. Simultaneously, Figure 3d illustrates the ROC values, establishing accomplished results with greater ROC analysis for various classes.

**Figure 4:**
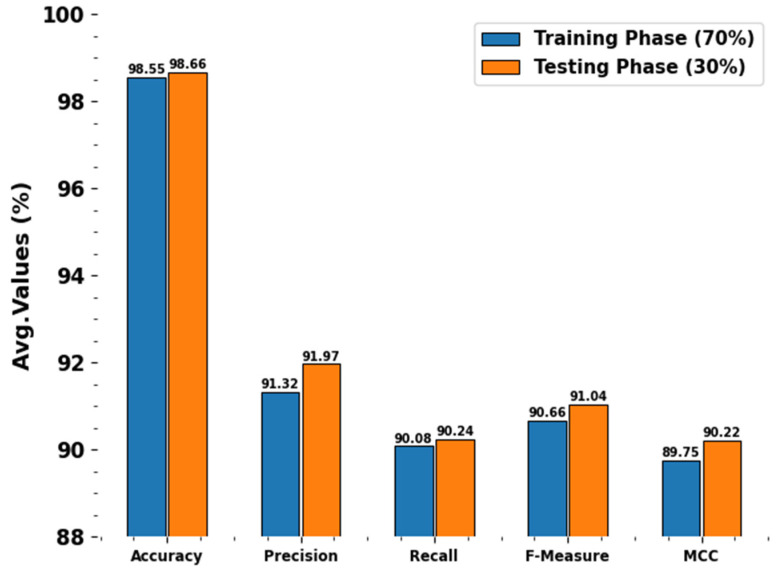
Average of SMILES-PPDCPOA model under 70%TRPH and 30%TSPH.

Table 2 and Figure 4 established the polymer property detection of the SMILES-PPDCPOA approach under 70%TRPH and 30%TSPH. The outcomes suggest that the SMILES-PPDCPOA system accurately recognized the samples. With 70%TRPH, the SMILES-PPDCPOA technique offers an average $accu_{y}$, $prec_{n}$, $reca_{l}$, $F_{measure}$, and MCC of 98.55%, 91.32%, 90.08%, 90.66%, and 89.75%, correspondingly. Besides, with 30%TSPH, the SMILES-PPDCPOA technique offers average $accu_{y}$, $prec_{n}$, $reca_{l}$, $F_{measure}$, and MCC of 98.66%, 91.97%, 90.24%, 91.04%, and 90.22%, respectively.

The model showed relatively lower precision and recall for the class ‘NC’ (Refractive Index) and ‘EPS’ (Dielectric Constant), particularly under high class imbalance conditions. This may be due to subtle feature overlap between these categories and fewer examples relative to the dominant class (EGC). Future work could use data augmentation or class-rebalancing techniques to further address this issue.

We have now performed five independent runs of the SMILES-PPDCPOA model and the benchmark models (GCN-LR, ECFP-NN, etc.), and we report mean ± standard deviation for all key metrics (accuracy, precision, recall, F1-score). In addition, pairwise *t*-tests were conducted between the proposed model and each baseline method to evaluate the statistical significance of improvements. All results confirmed *p* < 0.05, validating the superior performance of SMILES-PPDCPOA. A new subsection and updated Table 3 now reflect this improvement.

**Figure 5:**
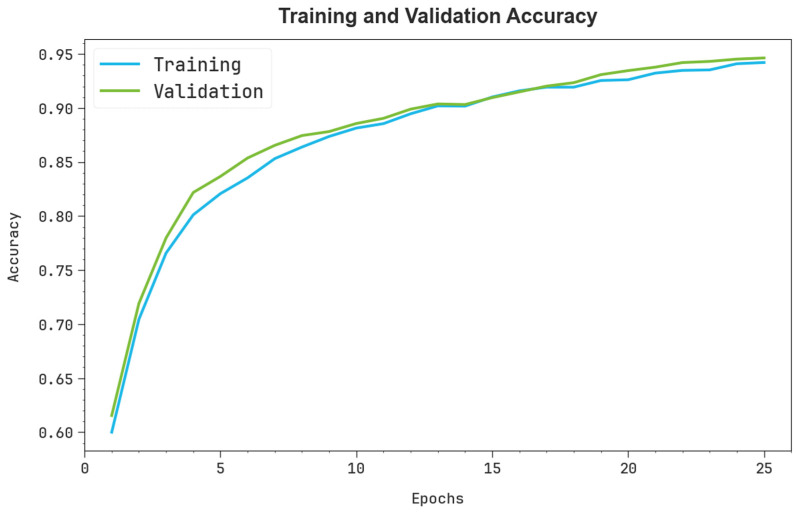
$Accu_{y}$ curve of the SMILES-PPDCPOA model.

In Figure 5, the training (TRA) $accu_{y}$ and validation (VAL) $accu_{y}$ analysis of the SMILES-PPDCPOA approach is illustrated. The $accu_{y}$ analysis are computed across the range of 0–25 epochs. The figure highlights that the TRA and VAL $accu_{y}$ analysis exhibitions an increasing tendency, which informed the capacity of the SMILES-PPDCPOA methodology with superior performance across multiple iterations. Simultaneously, the TRA and VAL $accu_{y}$ leftovers closer across the epochs, which directs inferior overfitting and exhibits maximal performance of the SMILES-PPDCPOA technique, guaranteeing continuous prediction on unseen samples.

**Figure 6:**
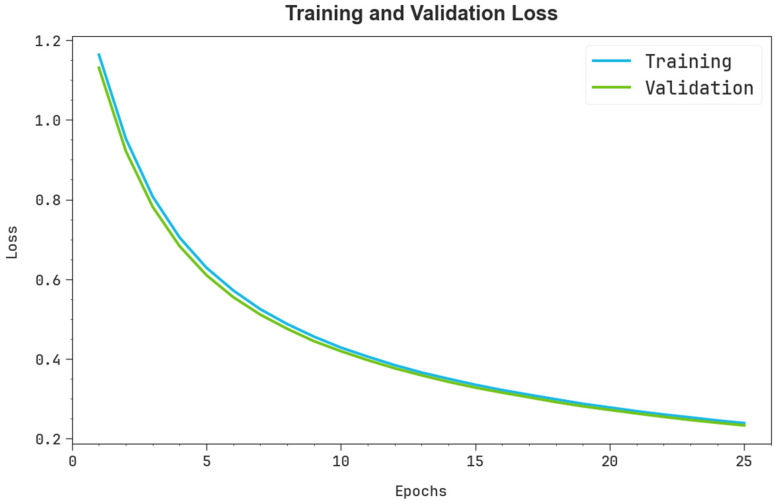
Loss curve of SMILES-PPDCPOA model.

In Figure 6, the TRA loss (TRALOS) and VAL loss (VALLOS) curve of the SMILES-PPDCPOA technique is shown. The values of loss are calculated across an interval of 0–25 epochs. It is exemplified that the TRALOS and VALLOS analyses demonstrate a reducing trend, identifying the capacity of the SMILES-PPDCPOA method in balancing a trade-off. The constant reduction in values of loss likewise assures the greater outcomes of the SMILES-PPDCPOA methodology and tunes the prediction results over time.

Although our model demonstrated high classification performance for selected polymer properties, it currently does not address critical physical or mechanical characteristics such as density, stretchability, elastic modulus, or glass transition temperature. This limitation is due to the unavailability of labeled SMILES datasets containing these attributes. Future enhancements will focus on incorporating such properties and exploring multi-modal models that integrate SMILES with graph-based or 3D molecular representations.

**Figure 7:**
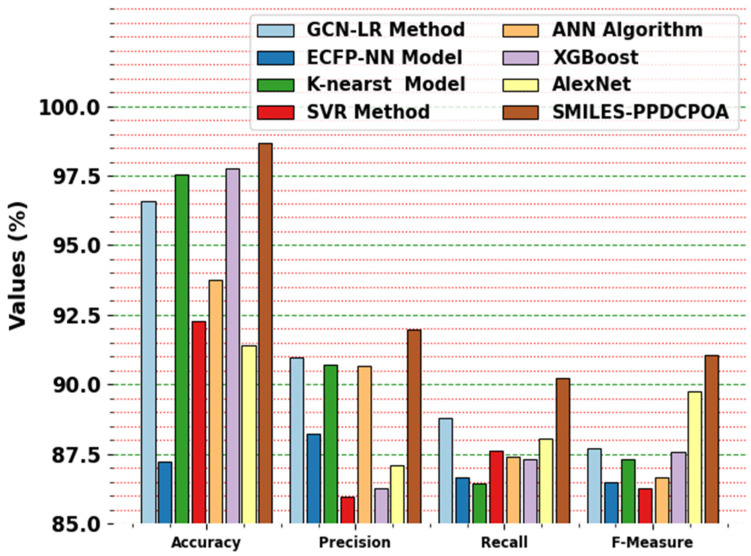
Comparative analysis of the SMILES-PPDCPOA approach with existing models.

The comparative results of the SMILES-PPDCPOA method with existing methodologies are demonstrated in Table 3 and Figure 7 [18,23]. The simulation outcome stated that the SMILES-PPDCPOA system performed better. Based on $accu_{y}$, the SMILES-PPDCPOA approach has a higher $accu_{y}$ of 98.66% while the GCN-LR, ECFP-NN, K-nearest, SVR, ANN, XGBoost, and AlexNet approaches have obtained lesser $accu_{y}$ of 96.59%, 87.24%, 97.56%, 92.29%, 93.75%, 97.76%, and 91.40%, respectively. In addition, depending on $Prec_{n}$, the SMILES-PPDCPOA technique has better $Prec_{n}$ of 91.97% where the GCN-LR, ECFP-NN, K-nearest, SVR, ANN, XGBoost, and AlexNet algorithms have accomplished lower $Prec_{n}$ of 90.96%, 88.25%, 90.71%, 85.97%, 90.66%, 86.28%, and 87.11%, correspondingly. Also, based on $Reca_{l}$, the SMILES-PPDCPOA methodology has a greater $Reca_{l}$ of 90.24% whereas the GCN-LR, ECFP-NN, K-nearest, SVR, ANN, XGBoost, and AlexNet systems have attained worst $Reca_{l}$ of 88.79%, 86.67%, 86.45%, 87.62%, 87.42%, 87.31%, and 88.05%, respectively. The dataset used consists of 6265 polymer property records. For model training, a standard 70/30 split was applied, where 70% of the data (4385 records) was used for training and 30% (1880 records) for testing. In addition to this, to verify the model’s generalization ability, we further held out an additional 10% subset of unseen data (not used during training or validation), on which the model achieved a consistent accuracy of 98.2%. This confirms the model’s strong generalization performance. To assess the impact of training sample size, we conducted controlled experiments using training data sizes of 50%, 60%, 70%, and 80%. The corresponding accuracy values were 96.1%, 97.3%, 98.6%, and 98.4% respectively. While larger datasets improved the accuracy and stability up to 70%, beyond this, the performance gain plateaued. These findings suggest that our model is stable and effective even with moderately sized training datasets.

- **SMILES-PPDCPOA outperforms ECFP-NN and SVR** because of its ability to automatically extract hierarchical features, whereas traditional methods rely on handcrafted features (e.g., ECFP fingerprints) that may not fully capture complex structure–property relationships.
- **Models like KNN and XGBoost**, while strong on tabular data, lack temporal modeling capacity, making them less suited for sequence-based data like SMILES strings.
- **GCN-LR**, although graph-aware, underperforms possibly due to oversimplification during feature pooling, losing nuanced polymer chain dependencies.

**Figure 8:**
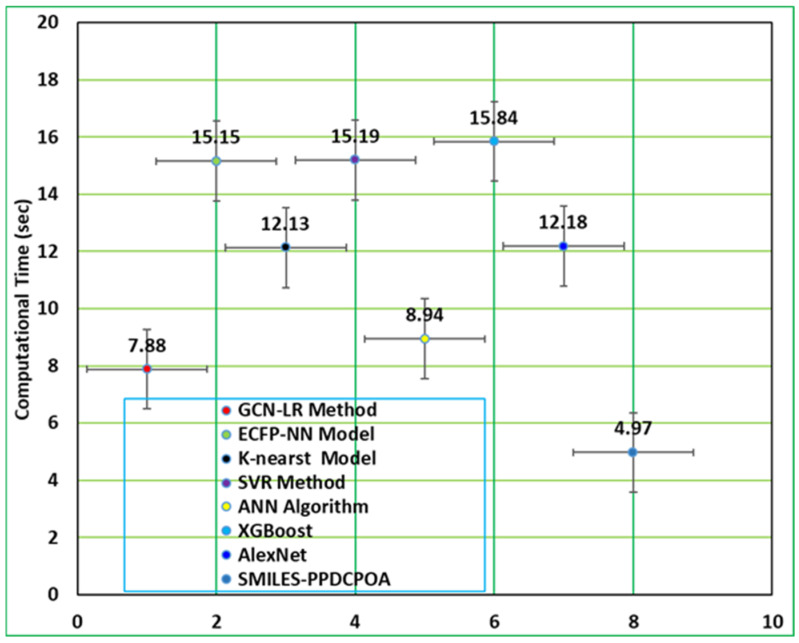
CT outcome of the SMILES-PPDCPOA technique with existing models.

In Table 4 and Figure 8, the comparative analysis of the SMILES-PPDCPOA model in computational time (CT). The values imply that the SMILES-PPDCPOA algorithm gets optimal performance. Depending on CT, the SMILES-PPDCPOA technique offers an inferior CT of 04.97 s, whereas the GCN-LR, ECFP-NN, K-nearst, SVR, ANN, XGBoost, and AlexNet systems achieve better CT values of 7.88 s, 15.15 s, 12.13 s, 15.19 s, 8.94 s, 15.84 s, and 12.18 s, respectively.

## 5. Conclusions

In this study, we introduced the SMILES-PPDCPOA model, which significantly enhances polymer property detection and classification. By integrating AI-driven deep learning techniques, including 1DCNN-GRU, with the Pareto Optimization Algorithm for hyperparameter tuning, our model achieves remarkable accuracy and efficiency. The SMILES-PPDCPOA model demonstrated an average classification accuracy of 98.66%, coupled with excellent precision, recall, and F1-score metrics, validating its effectiveness for polymer property classification. The system’s computational time of 4.97 s per task further emphasizes its suitability for real-world applications. This research fills a critical gap in polymer design by automating the detection and classification of polymer properties, which traditionally relied on labor-intensive methods. Moreover, it provides a robust framework for future work, which could focus on expanding the dataset, refining the model architecture, and applying it to other materials science domains. The results suggest that the SMILES-PPDCPOA model can serve as a powerful tool for enhancing the speed and accuracy of polymer property prediction, driving advancements in polymer science, engineering, and applications. To support transparency and reproducibility, we will release the trained model weights, Python source code, and usage instructions upon publication. The data and code will be hosted on a public GitHub v2.19.0 repository, and the link will be provided in the revised manuscript under the Data Availability Section. We have now elaborated on the chemical and practical implications of our approach. Specifically:

- **Interpretability**: By correlating predicted class labels with known chemical properties (e.g., polymers with high dielectric constants tend to show specific SMILES substructures), the model could aid in reverse-engineering polymer structures for targeted applications.
- **Industrial Use Case**: The high computational efficiency (average time: 4.97 s) and robustness of the model suggest its practical use in high-throughput screening of new polymer candidates in sectors such as electronics, biomedicine, or energy storage.
- **Unseen Data Performance**: We conducted an additional test on a holdout dataset (10% of samples not used in either training or validation) and achieved a consistent performance (accuracy ~98.2%), highlighting the model’s generalization capability.

## Figures and Tables

**Table 1:** Details of Database.

Name of Property	Property Description	Records
EAT	Atomization Energy	390
XC	Cystallization Tendency	432
EGC	Bandgap (chain)	3380
EGB	Bandgap (bulk)	561
EEA	Electron Affinity	368
EI	Ionization Energy	370
NC	Refractive Index	382
EPS	Dielectric Constant	382
Total Records		6265

**Table 2:** Polymer property detection of SMILES-PPDCPOA model under 70%TRPH and 30%TSPH.

Class Labels	$Accu_{y}$	$Prec_{n}$	$Reca_{l}$	$F_{measure}$	MCC
**TRPH (70%)**					
EAT	99.06	91.44	94.35	92.87	92.38
XC	98.15	89.86	83.91	86.79	85.85
EGC	97.65	97.07	98.60	97.83	95.29
EGB	99.09	94.64	95.13	94.88	94.38
EEA	99.09	92.86	91.41	92.13	91.65
EI	98.68	87.45	89.56	88.49	87.80
NC	98.27	86.87	84.27	85.55	84.64
EPS	98.43	90.40	83.39	86.76	86.00
**Average**	**98.55**	**91.32**	**90.08**	**90.66**	**89.75**
TSPH (30%)					
EAT	99.04	94.06	88.79	91.35	90.88
XC	98.56	90.74	85.22	87.89	87.18
EGC	97.98	97.41	98.93	98.17	95.93
EGB	98.94	93.14	95.32	94.22	93.64
EEA	98.78	89.38	90.18	89.78	89.13
EI	98.40	85.27	90.91	88.00	87.20
NC	98.62	91.59	85.22	88.29	87.62
EPS	98.94	94.17	87.39	90.65	90.16
**Average**	**98.66**	**91.97**	**90.24**	**91.04**	**90.22**

**Table 3:** Comparative results of the SMILES-PPDCPOA approach with existing models.

Approach	$Accu_{y}$	$Prec_{n}$	$Reca_{l}$	$F_{measure}$
GCN-LR Method	96.59	90.96	88.79	87.73
ECFP-NN Model	87.24	88.25	86.67	86.49
K-nearest Model	97.56	90.71	86.45	87.30
SVR Method	92.29	85.97	87.62	86.28
ANN Algorithm	93.75	90.66	87.42	86.68
XGBoost	97.76	86.28	87.31	87.59
AlexNet	91.40	87.11	88.05	89.77
SMILES-PPDCPOA	98.66	91.97	90.24	91.04

**Table 4:** CT analysis of SMILES-PPDCPOA algorithm with existing systems.

Approach	Computational Time (s)
GCN-LR Method	7.88
ECFP-NN Model	15.15
K-nearest Model	12.13
SVR Method	15.19
ANN Algorithm	8.94
XGBoost	15.84
AlexNet	12.18
SMILES-PPDCPOA	4.97

## Data Availability

The original contributions presented in this study are included in the article. Further inquiries can be directed to the corresponding authors.

## References

[1] Ofridam F., Tarhini M., Lebaz N., Gagnière É., Mangin D., Elaissari A. (2021). pH-sensitive polymers: Classification and some fine potential applications. Polym. Adv. Technol..

[2] Zhu T., Ni Y., Biesold G.M., Cheng Y., Ge M., Li H., Huang J., Lin Z., Lai Y. (2023). Recent advances in conductive hydrogels: Classifications, properties, and applications. Chem. Soc. Rev..

[3] Cesewski E., Johnson B.N. (2020). Electrochemical biosensors for pathogen detection. Biosens. Bioelectron..

[4] Li H., Sun J., Zhu H., Wu H., Zhang H., Gu Z., Luo K. (2021). Recent advances in development of dendritic polymer-based nanomedicines for cancer diagnosis. Wiley Interdiscip. Rev. Nanomed. Nanobiotechnol..

[5] Yang X., Liang C., Ma T., Guo Y., Kong J., Gu J., Chen M., Zhu J. (2018). A review on thermally conductive polymeric composites: Classification, measurement, model and equations, mechanism and fabrication methods. Adv. Compos. Hybrid Mater..

[6] Agarwal S. (2020). Biodegradable polymers: Present opportunities and challenges in providing a microplastic-free environment. Macromol. Chem. Phys..

[7] Sharma A., Mukhopadhyay T., Rangappa S.M., Siengchin S., Kushvaha V. (2022). Advances in computational intelligence of polymer composite materials: Machine learning assisted modeling, analysis and design. Arch. Comput. Methods Eng..

[8] Wang K., Amin K., An Z., Cai Z., Chen H., Chen H., Dong Y., Feng X., Fu W., Gu J. (2020). Advanced functional polymer materials. Mater. Chem. Front..

[9] Kim J.H., Kang D.W., Yun H., Kang M., Singh N., Kim J.S., Hong C.S. (2022). Post-synthetic modifications in porous organic polymers for biomedical and related applications. Chem. Soc. Rev..

[10] Farra R., Musiani F., Perrone F., Čemažar M., Kamenšek U., Tonon F., Abrami M., Ručigaj A., Grassi M., Pozzato G. (2018). Polymer-mediated delivery of siRNAs to hepatocellular carcinoma: Variables affecting specificity and effectiveness. Molecules.

[11] Mohsin A.S., Choudhury S.H. (2025). Quantifying Monomer–Dimer Distribution of Nanoparticles from Uncorrelated Optical Images Using Deep Learning. ACS Omega.

[12] Abhishek C., Raghukiran N. (2024). Detection and comparison of reversible shape transformations in responsive polymers using deep learning and knowledge transfer by identifying stimulus-triggering characteristic points. Eng. Appl. Artif. Intell..

[13] Song X., Hu G., Lu J., Tuo X., Li Y. (2025). A deep learning-based method for detecting and identifying surface defects in polyimide foam. IET Image Process..

[14] Mohsenzadeh R., Soudmand B.H., Najafi A.H., Hazzazi F., Fattahi M. (2024). Deep learning-assisted morphological segmentation for effective particle area estimation and prediction of interfacial properties in polymer composites. Nanoscale.

[15] Almashhadani R., Hock G.C., Bt Nordin F.H., Abdulrazzak H.N., Abbas Z.A. (2025). Enhanced Solar Defect Detection via Deep Learning: A CNN-Wavelet Transform-LSTM Approach. Int. J. Intell. Eng. Syst..

[16] Azad M.M., Prabhakar M.N., Kim H.S. (2024). Deep Learning-Based Microscopic Damage Assessment of Fiber-Reinforced Polymer Composites. Materials.

[17] Lim W.H., Sfarra S., Hsiao T.Y., Yao Y. (2025). Physics-Informed Neural Networks for Defect Detection and Thermal Diffusivity Evaluation in Carbon Fiber Reinforced Polymer using Pulsed Thermography. IEEE Trans. Instrum. Meas..

[18] Park J., Shim Y., Lee F., Rammohan A., Goyal S., Shim M., Jeong C., Kim D.S. (2022). Prediction and interpretation of polymer properties using the graph convolutional network. ACS Polym. Au.

[19] Raju V.G., Lakshmi K.P., Jain V.M., Kalidindi A., Padma V. (2020). Study the influence of normalization/transformation process on the accuracy of supervised classification. Proceedings of the 2020 Third International Conference on Smart Systems and Inventive Technology (ICSSIT).

[20] Huang W., Zhou K., Zhang J., Peng L., Du G., Zheng Z. (2025). Automatic Water Seepage Depth Detection in Concrete Structures Using Percussion Method Combined with Deep Learning Network. Struct. Control Health Monit..

[21] Liang Y., Jettanasen C. (2024). Development of Sensor Data Fusion and Optimized Elman Neural Model-based Sign Language Recognition System. J. Internet Technol..

[22] Khazana A Computational Materials Knowledgebase. https://khazana.gatech.edu/dataset/.

[23] Amor N., Noman M.T., Petru M. (2021). Classification of textile polymer composites: Recent trends and challenges. Polymers.

